# Introduction of software tools for epidemiological surveillance in infection control in Colombia

**Published:** 2015-06-30

**Authors:** Cristhian Hernández-Gómez, Gabriel Motoa, Marta Vallejo, Víctor M Blanco, Adriana Correa, Elsa de la Cadena, María Virginia Villegas

**Affiliations:** 1 Unidad de Resistencia Bacteriana y Epidemiología Hospitalaria, Centro Internacional de Entrenamiento e Investigaciones Médicas (CIDEIM), Cali, Colombia; 2 Departamento de Investigaciones, Universidad Pontificia Bolivariana, Medellín, Colombia

**Keywords:** Cross infection, drug resistance, microbial, epidemiological monitoring, software, quality assurance, health care, Colombia

## Abstract

**Introduction:**

Healthcare-Associated Infections (HAI) are a challenge for patient safety in the hospitals. Infection control committees (ICC) should follow CDC definitions when monitoring HAI. The handmade method of epidemiological surveillance (ES) may affect the sensitivity and specificity of the monitoring system, while electronic surveillance can improve the performance, quality and traceability of recorded information.

**Objective:**

To assess the implementation of a strategy for electronic surveillance of HAI, Bacterial Resistance and Antimicrobial Consumption by the ICC of 23 high-complexity clinics and hospitals in Colombia, during the period 2012-2013.

**Methods:**

An observational study evaluating the introduction of electronic tools in the ICC was performed; we evaluated the structure and operation of the ICC, the degree of incorporation of the software HAI Solutions and the adherence to record the required information.

**Results:**

Thirty-eight percent of hospitals (8/23) had active surveillance strategies with standard criteria of the CDC, and 87% of institutions adhered to the module of identification of cases using the HAI Solutions software. In contrast, compliance with the diligence of the risk factors for device-associated HAIs was 33%.

**Conclusions:**

The introduction of ES could achieve greater adherence to a model of active surveillance, standardized and prospective, helping to improve the validity and quality of the recorded information.

## Abstract (es)

**Introducción:**

Las infecciones asociadas a la atención en salud (IAAS) son un reto para la seguridad del paciente. Los comités de infecciones hospitalarios (CIH) deben realizar una vigilancia epidemiológica (VE) de las IAAS siguiendo los criterios de los Centros para el Control y Prevención de Enfermedades - EE.UU (CDC). La VE manual afecta la sensibilidad y especificidad del sistema de vigilancia, mientras que la VE electrónica mejora el desempeño, calidad y trazabilidad de la información registrada.

**Objetivo:**

Evaluar la implementación de una estrategia para la VE electrónica de las IAAS, resistencia bacteriana, consumo de antimicrobianos y características de los CIH en 23 clínicas y hospitales de alta complejidad en Colombia, en el periodo 2012-2013.

**Métodos:**

Se realizó un estudio observacional descriptivo de la introducción de herramientas informáticas en los CIH, evaluando la estructura y funcionamiento de los CIH, el grado de incorporación del software HAI Solutions y la cumplimiento al registro de la información requerida.

**Resultados:**

El 38% de las clínicas y hospitales (8/23) presentaron estrategias de vigilancia epidemiológica activa con criterios estándar del CDC. El 87% de las instituciones se adhirieron al módulo de captación de casos del software HAI Solutions, y el cumplimiento del diligenciamiento de los factores de riesgo de las IAAS asociadas a dispositivos fue del 33%.

**Conclusiones:**

La introducción del modelo de VE electrónica podría lograr un mayor cumplimiento a un modelo de vigilancia epidemiológica activo, estandarizado y prospectivo, contribuyendo al mejoramiento en la validez y calidad de la información registrada.

## Introduction

Healthcare-associated infections (HAI), previously known as nosocomial infections, are one of the biggest challenges in the care of critically ill patients in high-complexity hospitals. It is estimated that approximately 10% of patients worldwide develop at least one case of HAI during their hospital stay 1; HAI could be associated with additional healthcare costs between $ 12,000 and $ 35,000 per patient 2. The European Centre for Disease Prevention and Control (ECDC) established that 37,000 people die each year as a direct result of HAI 3. The ability to prevent and control HAI, coupled with reduction in transmission of multidrug resistant (MDR) microorganisms has become a standard assessment for organizations that oversee the quality assurance in healthcare and patient safety. Furthermore, these strategies must be incorporated into public health policies of a country 4.

In the seventies, the CDC established an active, prospective and systematic model of epidemiological surveillance (ES) of HAI. The infection control committees (ICC) are responsible for such surveillance through a proper collection, analysis and interpretation of clinical records, diagnostic aids and patient outcomes at discharge 5,6. Usually, HAI surveillance has been an arduous task; in many cases registration is handmade, which is incompatible with software, or a combination of both. This task of identification and collecting cases may occupy up to 45% of the total time in the workday of the ICC operating team 7,8. Although the consistency, continuity and the proper definition of cases are critical elements in the validity of the data reported to surveillance systems in public health, in many Latin American countries the information collected during HAI surveillance is incomplete or does not represent the actual state of hospitals. This fact indirectly reflects the level of development in infection control programs 9,10. In Colombia, the National Institute of Health (INS), in a pilot study conducted in 10 high-complexity clinics and hospitals during 2011, revealed the existence of heterogeneity in the development of surveillance programs and prevention and control of HAI. This situation could affect the ability to draw comparisons between the indicators reported by these institutions 11. In addition, the monitoring of HAI caused by MDR bacteria is a crucial element of any infection control program; however, traditional approaches to this activity have shown limited scope and effectiveness in data consolidation 12. Nowdays, progress in information technology in hospital settings has been applied mainly in recording and storage of information in the physician-patient encounter, which has led to tremendous optimization in the management of results and medical orders 13,14.

The ICC recognize that electronic records of data may improve the efficiency and accuracy for monitoring HAI, moreover, it could allow monitoring MDR bacteria, comparing rates of device-associated HAI between hospital services, and provide effective measures in order to strengthen the prevention and control strategies from the perspective of the different healthcare and administrative actors 13,15.

The Association for Professionals in Infection Control and Epidemiology (APIC) states that development of new methods of surveillance and significant indicators for measuring the HAI must be a priority in its strategic plan projected for 2020 16. In a systematic review, Leal and Laupland reported that using electronic surveillance methods may reduce 61% of the time required for this activity 16 and could be used in education and prevention.

The limited resources of the ICC require prioritization of tasks, especially surveillance. The electronic transfer of data between hospitals and the public health information system in a country may produce significant benefits for surveillance of HAI, bacterial resistance (BR) and consumption of antimicrobials (CA); the efficiency, timeliness, comprehensiveness and drivability of the information recorded during electronic surveillance are the pillars that provide the introduction of software in ICC 17.

The aim of this study was to evaluate the characteristics of ICC in hospitals and implement a strategy of ES of HAI-BR-CA in 23 high-complexity clinics and hospitals in Colombia during 2012-2013.

##  Materials and Methods

An observational study was conducted in 23 high-complexity clinics and hospitals located in 10 capital cities in Colombia. These institutions are part of the National Network of bacterial resistance and HAI of CIDEIM. This study was conducted in three stages: situational assessment, introduction of information technologies, and evaluation and sharing of findings to the institutions.

###  Situational assessment

During 2012, we visited the ICC of clinics and hospitals in the network to assess the ES program. To do this, the Rapid Evaluation guide for hospital programs for prevention and control of nosocomial infections by the Pan American Health Organization (PAHO), version 2011 18 was used. Before the verification visit, the institutions filled out an evaluative questionnaire that included questions about general characteristics of the clinic or hospital, the ICC organization, the use of a manual for standard operating procedures for the diagnosis of HAI, and socialization of results to healthcare workers. During the verification visit, we studied the protocols, guidelines and manuals with which the ICC are managed in each institution. Furthermore, we accompany the ES routine operational processes of the ICC, microbiology laboratory and pharmacy. HAI case definitions should correspond to those established by the CDC according to regulations by the INS in 2012 19. Subsequently, the institutional situational assessment report and the improvement options were presented (Table 1).

###  Introduction of information technology (IT) 

First, clinics and hospitals adopted the minimum requirements for the incorporation of the software to the ES strategy, including a scheme of active, prospective, systematic and standard ES, according to the CDC definitions. Then ICC staff was trained in the use of the Healthcare Associated Infections Solutions (HAI Solutions^®)^ software, which was installed on a mobile tablet-type that was delivered to the ICC in each institution. As part of the training, the introduction of the device handling, the software interface and the capture and storage of data were performed; additionally, the synchronization frequency of the software to the server was defined according to the ES program in each ICC.

The HAI Solutions^®^ data collection module adopts and integrates dynamically, criterion to criterion, different types of variables as the definitions of National Healthcare Safety Network (NHSN, version 2012) for surveillance and monitoring of HAI, BR and CA 20: device-associated HAI (central venous catheter-associated bloodstream infection, symptomatic catheter-associated urinary tract infection and ventilator-associated pneumonia) and surgical site infections (SSI) related with surgical specialties (neurosurgery, cardiovascular and orthopedics); through daily collection of device-day information (days of mechanical ventilation, indwelling catheter and/or central venous catheter), and surgical risk index (SRI). Additionally, the module includes information of the isolated microorganism, device-days of exposure for patients, the rate of surgical risk for the SSI, and the form for recording consumption of antimicrobials through the Defined Daily Dose (DDD) methodology established by the World Health Organization. The DDD is a technical unit of measurement that relates the consumption of a drug over a period of time according to the occupancy rate and bed availability for the service of interest. Thus, the DDD reference to the drug will be the average daily dose of a drug used on its main indication in a given dosage form and in adults 21.

Between July 2012 and June 2013, after standardizing ES processes, cases of device-associated HAI and SSI reported in intensive care units were registered in the software. Each case contained socio-demographic data of patients, the criteria for case definition according to CDC-NSHN, microbiological data and outcomes at hospital discharge.

Finally, the communication channels for socializing needs of information presented by the institutions were established. HAI cases and denominators to estimate rates of infection were registered. The quality of the information recorded and the time of loading the data to the server were also evaluated at this stage.

### Evaluation and feedback of the use of IT

Each institution received a report about the accomplishment of the Rapid Evaluation guide for hospital programs for prevention and control of nosocomial infection of the PAHO 18 and an improvement plan was defined.

An analysis of compliance frequencies of each criterion evaluated in the ICC diagnosis and also the rate of fulfillment in the processing of cases through the HAI Solutions^®^ software was performed.

## Results

### Diagnosis

Of the 23 clinics and hospitals evaluated, 98% had official documents recognizing the existence of the ICC in the organizational chart and established roles in surveillance, prevention and control of HAI-BR-CA. All institutions had ES strategies within the main activities of ICC; 91% had between one and two nurses available to collect cases of HAI during ES of them, mainly in intensive care units, while 56% also had technical nursing staff for monitoring general wards (Table 1). All ICC mentioned maintained direct and constant communication with support services and diagnostic aids for the study of cases.

**Table 1.**
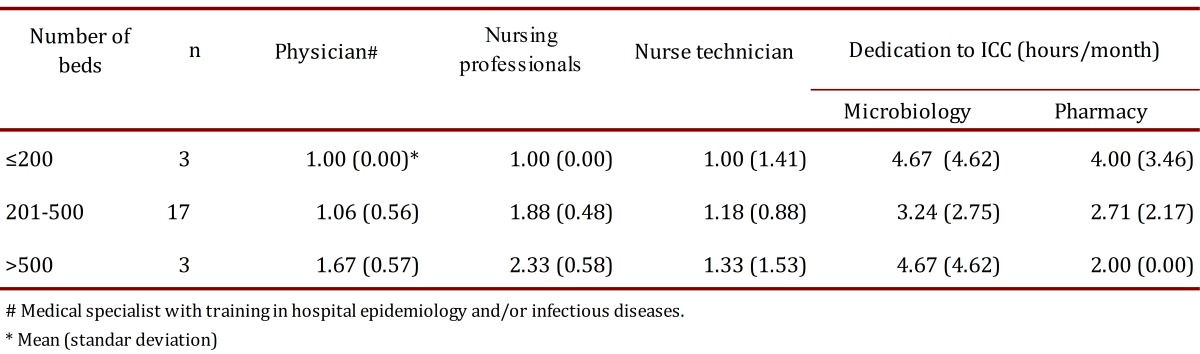
Characterization of ICC in 23 high-complexity clinics and hospitals located at 10 Colombian cities.

Eighty-seven percent (20/23) of institutions reported conducting the ES of the HAI using the criteria of NHSN-CDC, 14% had HAI definitions published in 2011, 71% in 2010 and the remaining 15% continued with 2009 or earlier definitions. Of the 20 institutions that performed the ES with NHSN-CDC criteria, 30% presented definitions of medical associations or research groups that included changes in the criteria of device-associated HAI. Simultaneously, during the verification visit, 57% of institutions that adopted the NHSN-CDC criteria from the original source included institutional changes for the interpretation of those criteria.

Of all institutions, only 22% implemented active ES, 65% combined strategies of active and passive surveillance, and 13% were only passive ES. Seventy-three percent of the institutions reported that the ES strategy appeared more passive when watching the SSI.

### Introduction of technologies

The period of implementation of the data collection module HAI Solutions^®^ took three months for the 20 institutions that actively practiced ES of device-associated HAI using the unchanged CDC-NHSN definitions or those that adapted their ES strategy according to operating requirements.

In the study period, 1,114 cases of device-associated HAI and SSI reported in intensive care units were registered.

Regarding the daily collection of device-days, only 8 institutions complied with the daily log in the software, while 12 institutions conducted the collection and recording of information from data consolidated in handmade templates. The reasons for non-compliance in the daily register of device-days were explained and several factors were found such as: temporary absence of ICC staff during that month, more than one person recording data by each hospital service with a single device capturing the entire institution, and the need for double data entry as it should also be registered in the statistics collection systems of medical records in specific lines for each institution (Table 2).

**Table 2.**
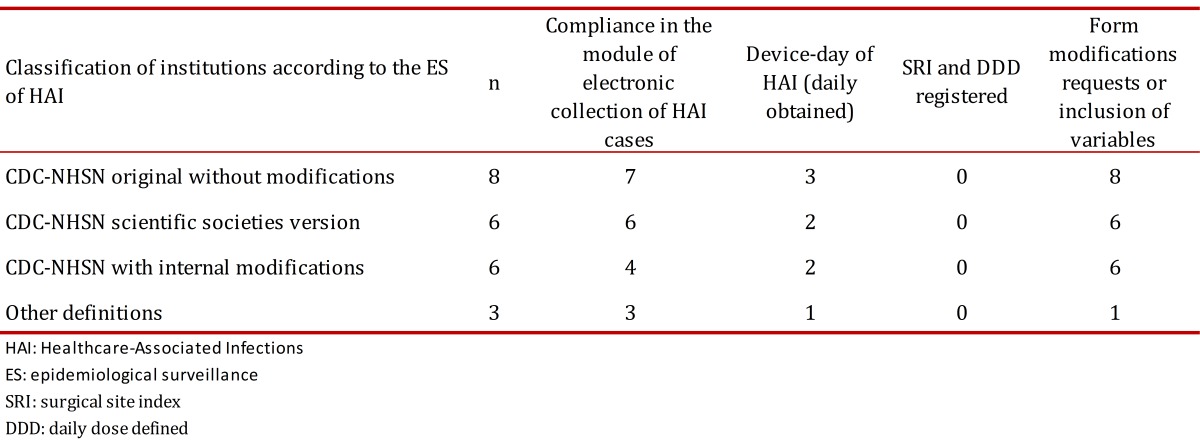
Fulfillment of HAI Solutions software introduction in ES of HAI in 23 clinics and hospitals located at 10 Colombian cities.

During the period under review, the institutions did not comply with the registration of SRI for stratification of rates in SSI; the main reason for non-compliance to the process was the absence of information required to calculate the SRI in patients undergoing surgery during daily sessions of surveillance. This made difficult to capture data and delayed the opportunity to identify and analyze cases.

With regard to DDD, only 10% of institutions reported antibiotic consumption with DDD methodology and data recording was not continuous during the period evaluated. The main obstacle to this was the consolidation of all information required for the processing or limited access to the device by the Central Pharmaceutical (Table 2).

## Discussion

The present study demonstrates that the evaluated hospitals showed variation between what was reported by the ICC and what was found in the follow-up visits in the ES of HAI, with a number of professionals in the ICC lower than the minimum required. There were also difficulties in standardizing HAI criteria.

Therefore, it is necessary to strengthen ES programs before introducing computer tools for ES in the IC; it is required to have a larger number of professionals trained or specialized in infectious diseases and infection as part of the ICC 8. According to the SENIC project (in 1976), it is necessary to have at least one nurse for every 250 beds, a microbiologist and a referent in infectious diseases; with this proportion was demonstrated that HAI could be reduced as low as 32% 22. The ICC evaluated on our network preserved this relation, having up 2.3 professionals in institutions with more than 500 beds; however with changes in the health care system currently involving complex care of patients in acute care institutions, the functions and obligations has increased; and therefore, the responsibilities of nurses in ICC need to be closely considered. For all of these reasons, it should be required to increase the ratio of professionals by number of beds in institutions with high rates of HAI, in order to be at least 1 person per 100 beds 23.

Since most clinics and hospitals combined different surveillance strategies, the awareness of the staff promoting standardized, active, prospective and systematic strategies in accordance with the CDC-NHSN terms of reference is crucial in order to be a critical element contributing to the validity and traceability of prevention and control measures based on the data collected.

Although a strategy of ES contributes to reduce sources of error in these cases, it is not possible to ensure the success of the institutional ES if the institutional processes to support and guarantee the quality of the captured information are not included 24-26. Once the quality of information is achieved, internal or external evaluation of comparative HAI and RB rates will identify the strengths and weaknesses of strategies for prevention and control, stimulate inter-service and inter-institutional competitiveness and set the value of interventions aimed at reducing HAI. A first step will be to avoid changes in the CDC-NHSN definitions, which were changed in 57% of the evaluated institutions; standardization will allow internal/external comparison and analysis, according to reports from Villalobos *et al* 11,27,28.

With regard to the introduction of IT in surveillance programs and prevention and control of HAI and RB, the use of software in hospitals must overcome three major challenges to demonstrate its operational value. The first is to transmit data in standard but flexible formats to meet the specific needs of users, the second challenge will be the normalization of variables according to the codification of the computer systems of users without losing the reliability of the information recorded, and third, ensuring the quality of information transmitted, guaranteeing that this data truly reflect what users captured thorough their sources of information as they go recording cases. Although HAI Solutions(r) software incorporates automatic processing of data strategies in the device 29,30, and in our study, 87% of institutions met capture and record through the collection module case by HAI Solutions software, it is required to evolve into an automated surveillance HAI system with 100% sensitivity for the detection of cases, which allow the staff to focus and discard those false positive cases 31.

In short, the introduction of software tools for the ES of HAI is a significant advance in ensuring the quality of the information recorded, however, awareness of the institutions and the creation of policies to support the number of professionals required to the ICC, adequate staff training and awareness of the rigor required in the ES of HAI is a fundamental step that every institution must do before adopting this type of technology.

## Conclusions

This study shows that a significant proportion of the evaluated Colombian hospitals lacks standardized surveillance of HAI, especially in the implementation of the CDC criteria for the proper definition of cases of HAI methods, which limits the comparison between them. The standardized surveillance of HAIs is important to ensure the reliability and quality of the data, so appropriate strategies can be implemented to prevent and control infections nationwide. Surveillance systems should verify these conditions before adopting computer data or report to the ES of the HAI tools.

## References

[1] WHO The burden of health care-associated infection worldwide.

[2] El-Masri MM, Oldfield MP (2012). Exploring the influence of enforcing infection control directives on the risk of developing healthcare associated infections in the intensive care unita retrospective study. Intensive Crit Care Nurs.

[3] Martin M, Zingg W, Hansen S, Gastmeier P, Wu AW, Pittet D (2013). Public reporting of healthcare-associated infection data in EuropeWhat are the views of infection prevention opinion leaders?. J Hosp Infect.

[4] Greene LR, Cain TA, Khoury R, Krystofiak SP, Patrick M, Streed S (2009). APIC position paper The importance of surveillance technologies in the prevention of health care-associated infections. Am J Infect Control.

[5] Hebden JN (2012). Rationale for accuracy and consistency in applying standardized definitions for surveillance of health care-associated infections. Am J Infect Control.

[6] Mertens K, Morales I, Catry B (2013). Infections acquired in intensive care unitsresults of national surveillance in Belgium, 1997-2010. J Hosp Infect.

[7] Grota PG, Stone PW, Jordan S, Pogorzelska M, Larson E (2010). Electronic surveillance systems in infection preventionorganizational support, program characteristics, and user satisfaction. Am J Infect Control.

[8] Woeltje KF (2013). Moving into the futureelectronic surveillance for healthcare-associated infections. J Hosp Infect.

[9] Perla RJ, Peden CJ, Goldmann D, Lloyd R (2009). Health care-associated infection reportingthe need for ongoing reliability and validity assessment. Am J Infect Control.

[10]  Acosta-Gnass S,  Aragon JC,  Benoit SR,  Betancourt MI (2008). Evaluación de la infección hospitalaria en siete países latinoamericanos. Rev Panam Infectol.

[11] Villalobos AP, Barrero LI, Rivera SM, Ovalle MV, Valera DA (2014). Surveillance of healthcare associated infections, bacterial resistance and antibiotic consumption in high-complexity hospitals in Colombia, 2011. Biomédica.

[12] Shaban-Nejad A, Riazanov A, Charland KML, Rose GW, Baker CJO, Tamblyn R (2012). HAIKUA Semantic Framework for Surveillance of Healthcare-Associated Infections. Procedia Comput Sci.

[13] Trick WE (2008). Building a data warehouse for infection control. Am J Infect Control.

[14] Cartmill RS, Walker JM, Blosky MA, Brown RL, Djurkovic S, Dunham DB (2012). Impact of electronic order management on the timeliness of antibiotic administration in critical care patients. Int J Med Inform.

[15] Wright M-O, Carter E, Pogorzelska M, Murphy C, Hanchett M, Stone PW (2012). The APIC research agendaresults from a national survey. Am J Infect Control.

[16] Leal J, Laupland KB (2008). Validity of electronic surveillance systemsa systematic review. J Hosp Infect.

[17] Vozikis A (2009). Information management of medical errors in GreeceThe MERIS proposal. Int J Inf Manage.

[18] Organización Panamericana de Salud (2011). Guía de evaluación rápida de programas hospitalarios en prevención y control de las infecciones asociadas a la atención de salud.

[19] Instituto Nacional de Salud (2012). Implementación de la estrategia de vigilancia en salud pública de infecciones asociadas a la atención en salud.

[20] CDC Tracking Infections in Acute Care Hospitals/Facilities - NHSN.

[21] Lallana AMJ, Feja SC, Malo FS, Abad DJM, Bjerrum L, Rabanaque HMJ (2012). Variations in the Prescription of Antibiotics among Primary Care Areas in the Autonomous Region of Aragon, Spain. Rev Esp Salud Publica.

[22] Haley RW, Quade D, Freeman HE, Bennett JV (1980). The SENIC ProjectStudy on the efficacy of nosocomial infection control (SENIC Project). Summary of study design. Am J Epidemiol.

[23] Kramer A, Assadian O, Helfrich J, Krüger C, Pfenning I, Ryll S (2013). Questionnaire-based survey on structural quality of hospitals and nursing homes for the elderly, their staffing with infection control personal, and implementation of infection control measures in Germany. GMS Hyg Infect Control.

[24] Freeman R, Moore LSP, García Álvarez L, Charlett A, Holmes A (2013). Advances in electronic surveillance for healthcare-associated infections in the 21st Centurya systematic review. J Hosp Infect.

[25] Keller SC, Linkin DR, Fishman NO, Lautenbach E (2013). Variations in identification of healthcare-associated infections. Infect Control Hosp Epidemiol.

[26] Lin MY, Hota B, Khan YM, Woeltje KF, Borlawsky TB, Doherty JA (2010). Quality of traditional surveillance for public reporting of nosocomial bloodstream infection rates. JAMA.

[27] El-Saed A, Balkhy HH, Weber DJ (2013). Benchmarking local healthcare-associated infectionsavailable benchmarks and interpretation challenges. J Infect Public Health.

[28] Feltovich F, Fabrey LJ (2010). The current practice of infection prevention as demonstrated by the practice analysis survey of the Certification Board of Infection Control and Epidemiology, Inc. Am J Infect Control.

[29] Edwards JR, Pollock DA, Kupronis BA, Li W, Tolson JS, Peterson KD (2008). Making use of electronic datathe National Healthcare Safety Network e Surveillance Initiative. Am J Infect Control.

[30] Turner C, Bishay H, Bastien G, Peng B, Phillips RC (2007). Configuring policies in public health applications. Expert Syst Appl.

[31] Chiou S-F, Chuang J-H (2011). Only automated surveillance with 100% sensitivity can save ICPs' time. Am J Infect Control.

